# CCN2 functions as a modulator of cell cycle regulation in human dermal fibroblasts

**DOI:** 10.1002/ccs3.70003

**Published:** 2025-02-01

**Authors:** Taihao Quan, Yuan Shao, Trupta Purohit, Yiou Jiang, Zhaoping Qin, Gary J. Fisher, Nathan H. Lents, Joseph J. Baldassare

**Affiliations:** ^1^ Department of Dermatology University of Michigan Medical School Ann Arbor Michigan USA; ^2^ Department of Pharmacological Sciences at Saint Louis University St. Louis Missouri USA

**Keywords:** CCN2, cell cycle, fibroblast

## Abstract

CCN2 is widely regarded as a profibrotic factor involved in fibrotic disorders by regulating extracellular matrix (ECM). We report here that CCN2 functions as a critical cell cycle regulator in primary human dermal fibroblasts (HDFs). siRNA‐mediated knockdown of CCN2 halted proliferation of primary HDFs, which was rescued by a siRNA‐resistant CCN2 expression vector. Furthermore, CCN2 knockdown caused a significant accumulation of cells in G1/G0 phase and blocked entry into S‐phase. Mechanistically, CCN2 knockdown blocked cyclin E and CDK4/cyclin D nuclear translocation, and abrogated CDK2 activity. Markedly, CCN2 translocated to the nucleus and co‐localized with cyclin D1 upon cell cycle stimulation. Finally, we show that CCN2, a bona fide YAP/TAZ target gene, partially mediates YAP/TAZ‐dependent proliferation of primary HDFs. These data provide evidence of a novel CCN2 function as a cell cycle regulator in primary HDFs proliferation, in addition to its known role in ECM regulation.

## Graphical abstract

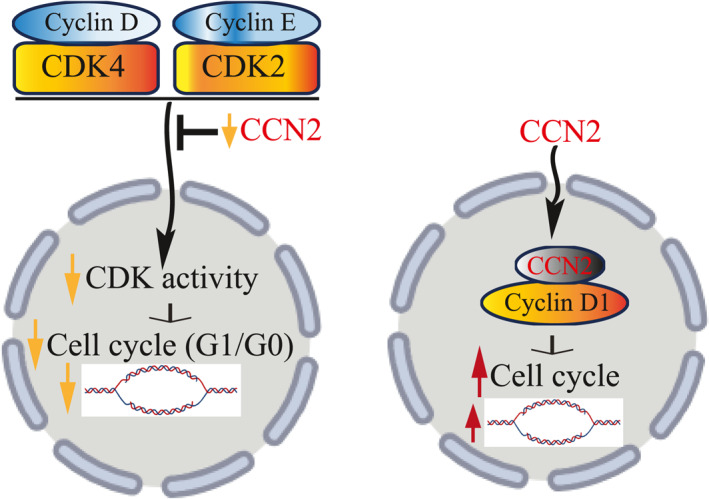

## 1 INTRODUCTION

CCN2, also known as connective tissue growth factor (CTGF), is a matricellular protein of the cell communication network (CCN) family of extracellular matrix‐associated proteins.^1^, ^2^ CCN2 is the second member of the CCN family of proteins, which consists of six members, numbered from CCN1 to CCN6.^3^ CCN2 exhibits diverse biological activities in vitro, including cell adhesion, migration, and extracellular matrix (ECM) production.^4^, ^5^, ^6^ Over the past decade, the role of CCN2 in fibrotic disorders has been increasingly studied. CCN2 is often elevated in fibrotic conditions affecting the skin, lungs, and kidneys, establishing CCN2 as a recognized marker of fibrosis.^7^ It is believed to stimulate excessive collagen production in these disorders.^8^, ^9^, ^10^ The role of CCN2 in collagen production was further elucidated through animal studies. In CCN2 transgenic mice, selective expression of CCN2 in fibroblasts promoted systemic tissue fibrosis.^10^ Additionally, CCN2 injection into mouse skin induced fibrosis by stimulating collagenous ECM synthesis.^11^ Conversely, CCN2‐null mice die shortly after birth, primarily due to skeletal defects.^12^ This observation underscores the crucial role of CCN2 in regulating cartilage ECM during development. Collectively, these observations elucidate the pleiotropic functions of CCN2 in physiological and pathological contexts, with particular emphasis on its pivotal role in ECM homeostasis and fibrotic pathogenesis. In human skin dermal fibroblasts, CCN2 is induced by transforming growth factor‐β (TGF‐β) and acts as a downstream mediator of TGF‐β stimulated collagen synthesis.^13^ Interestingly, CCN2 exhibits contrasting regulation in different contexts. Although it is upregulated in fibrotic disease, CCN2 is downregulated in fibroblasts of aged human skin, leading to reduction in production of collagen and contribution to dermal aging.^13^, ^14^, ^15^ Although the role of CCN2 in collagenous ECM synthesis is recognized,^16^, ^17^ its complete biological functions remain to be fully elucidated.

CCN2 is known to promote proliferation in various cell types, such as fibroblasts, chondrocytes, osteoblasts, mesangial cells, and hepatic stellate cells.^7^, ^9^ In the skin, CCN2 stimulates proliferation of fibroblasts, particularly in the context of wound healing and fibrosis. These proliferative effects are often mediated through interactions with various growth factors and cell surface receptors, including integrins and TGF‐β signaling pathways.

We report here that CCN2 functions as a novel cell cycle regulator essential for primary human dermal fibroblast (HDF) proliferation. The cell cycle is fundamental in biology, playing critical roles in growth, development, cellular homeostasis, tissue repair/regeneration, genetic stability, and cancer.^18^ In the skin, cell cycle maintains homeostasis and function while influencing various skin diseases.^19^ The cell cycle is crucial for epidermal integrity, regulating keratinocyte proliferation and differentiation. In the dermis, it ensures fibroblasts proliferate as needed to maintain skin structure and function. For example, during wound healing, the cell cycle activates in both skin epidermal and dermal cells, promoting rapid division and migration to the wound site.

The progression of the cell cycle is precisely controlled, with various checkpoint proteins regulating the transition between its distinct phases.^20^ CCND proteins, also known as cyclin D proteins, are important regulators of the cell cycle in eukaryotic cells. There are three main types of CCND protiens in mammals: Cyclin D1, D2, and D3 (encoded by CCND1, CCND2, and CCND3 genes, respectively). CCND proteins play a crucial role in controlling the G1 to S phase transition of the cell cycle. In addition, a group of enzymes known as cyclin‐dependent kinases (CDKs) are essential for orchestrating the progression of the cell cycle through their protein phosphorylation activity. There are multiple CDKs, numbered CDK1 through CDK20, but not all are involved in cell cycle regulation. CDK1, CDK2, and CDK4/6 play major roles in controlling the cell cycle. CDK inhibitors (CKIs) are proteins that regulate the activity of CDKs. CKIs negatively regulate CDK activity, helping to control cell cycle progression and prevent uncontrolled cell division. There are two main families of CKI proteins: a) INK4 family: p16, p15, p18, p19 and b) Cip/Kip family: p21, p27, p57. Our study found that CCN2 knockdown increased cells in G1/G0 phase while significantly blocking S‐phase entry. Mechanistically, CCN2 knockdown inhibited cyclin E and CDK4/cyclin D nuclear translocation and abrogated CDK2 activity. Unexpectedly, we found that CCN2 itself translocated to the nucleus and co‐localized with cyclin D1 when cell cycle activated. Although CCN2 is primarily recognized for its extracellular functions in ECM regulation, its intracellular roles, especially in the nucleus, remain largely unexplored. These findings highlight a previously unknown function of CCN2 as a regulator of the cell cycle in primary HDF proliferation. This expands our understanding of CCN2 beyond its well‐established role in ECM production, suggesting a more complex and multifaceted involvement in cellular processes.

## 2 MATERIAL AND METHODS

### 2.1 Cell culture and transient transfections

Primary human HDFs were isolated from normal human skin biopsies, as described previously.^21^ Briefly, full‐thickness punch biopsies were obtained from human volunteers and dermis was separated from epidermis by trypsinization (0.25% trypsin, 0.1% EDTA) of the tissue for 30 min at 37°C in phosphate‐buffered saline (PBS) solution. The biopsy was minced into small pieces with a scissors and forceps, and tissue fragments were placed on a small dish. Dulbecco's Modified Eagle's Medium (DMEM) containing 4.5 g/L glucose and 2 mM l‐glutamine (BioWhittaker, Walkersville, MD), supplemented with 10% (vol/vol) fetal calf serum (FCS) (Invitrogen, Carlsbad, CA). All media were supplemented with 100 IU/mL penicillin and 0.1 mg/mL streptomycin. Only a minimal amount of medium was included so that tissue pieces would adhere to the plastic surface. The dishes were maintained at 37°C in an atmosphere of 95% air and 5% CO_2_. The tissue was removed after 5–7 days, at which time cells were migrated out from the edge of the tissue fragments. Cells of the two to nine passages in actively growing condition were used for experiments. Transfections were carried as previously described.^22^, ^23^ Transient transfection of emerald green fluorescent protein (EmGFP, The Vivid Colors™ ThermoFisher ‎Waltham, MA) indicated that the transfection efficiency can be up to 80% in human primary skin fibroblasts by electroporation.^23^ 1 × 10^6^ primary HDF cells were electroporated with siRNAs or plasmid DNA. CCN2 siRNA was designed based on human mRNA open reading frames according to the OligoEngine (Seattle, WA) website instructions and submitted to a BLAST search against human genome database to confirm specificity. siRNAs for YAP (GACATCTTCTGGTCAGAGA) and TAZ (AGGTACTTCCTCAATCACA) were purchased from Sigma Chemical Company (St. Louis, MO). Control siRNA (AATTGTCCGAACGTGTCACGT) were purchased from Qiagen (Chatsworth, CA). A CCN2 expression plasmid was created via site‐directed mutagenesis to confer resistance to CCN2 siRNA.

### 2.2 Cell cycle synchronization

Cells were cultured in DMEM supplemented with serum for 17 h. To synchronize the cells in G0, subconfluent HDFs were growth‐arrested using the following procedure: cells were washed once with phenol red‐free α‐minimal essential medium (Invitrogen, CA) containing 2 mM l‐glutamine (Bio‐Whittaker), then incubated in this same medium for 4 days. After this serum arrest period, cell cycle re‐entry was stimulated by adding 15% serum (Invitrogen, Carlsbad, CA) for the specified durations.

### 2.3 RNA isolation and quantitative real‐time RT‐PCR

Total cellular RNAs were isolated using TRIzol reagent (Invitrogen, CA) following the manufacturer's instructions. Reverse transcription of 100 ng total RNA was performed using TaqMan reverse transcription kit (Applied Biosystems, Carlsbad, CA, USA) to obtain cDNA for PCR templates. TaqMan universal PCR master mix reagents (Applied Biosystems, Carlsbad, CA, USA) were used for real‐time PCR on a 7700 sequence detector (Applied Biosystems, Carlsbad, CA, USA). The real‐time PCR primers were obtained from RealTimePrimers.com (Real Time Primers, LLC, Elkins Park, PA). The housekeeping gene 36B4 was used as an internal control to quantify the target gene mRNA expression levels.

### 2.4 Cytosol and nuclear proteins fractionation

Primary HDFs were washed with PBS and harvested with 0.05% trypsin for 4 min at 37°C. Cytosol and nuclear proteins were obtained by preparing cell lysate fractions. The cells were lysed in whole cell extraction buffer (25 mM HEPES (pH 7.7), 0.3 M NaCl, 1.5 mM MgCl2, 0.2 mM EDTA, 0.1% Triton X‐100, 0.5 mM DTT, 20 mM β‐glycerolphosphate, 0.1 mM Na_3_VO_4_, 2 μg/mL leupeptin, and 100 μg/mL PMSF). The resulting lysate was centrifuged at 1,300 × g for 5 min to pellet nuclei. The nuclei pellets were then resuspended in lysis buffer and sonicated, whereas the supernatants were centrifuged at 14,000 × g to remove any contaminating nuclei. Cytosol and nuclear proteins were distinguished by western blot analysis, which showed the presence of hexokinase I or SOD (Santa Cruz Biotechnology, CA) and the absence of histone lamin A/C or PARP (Santa Cruz Biotechnology, CA) in the cytosolic fraction.

### 2.5 Western blot analysis

Whole cell proteins were lysed in whole cell extraction buffer, as described above. Protein concentrations were determined by Coomassie Plus (Pierce Protein Biology, Thermo Fisher Scientific, MA) as recommended by the manufacturer. Equal amounts of protein (∼40 μg/lane) were loaded onto 6%–12% gradient sodium dodecyl sulfate‐polyacrylamide (SDS) gels, which were then transferred onto polyvinylidene difluoride membranes and blocked with PBST (0.1% Tween 20 in PBS) containing 5% nonfat milk for one hour at room temperature. Collect the cell culture media from cells grown under the desired conditions. Cell culture media was collected and concentrated in order to detect secreted CCN2. Primary antibodies were incubated for 1 hour at room temperature. The following antibodies were used for Western blotting: CCN2, LaminA/C, SOD, and PARP (Santa Cruz Biotechnology, CA); p‐H1, CDk2, CDk4, pRb, and cylin D1 (Cell signaling Technology, MA); p‐ERK1/2 and p‐Akt (Waltham, MA); β‐actin (Sigma, St. Louis, MO). CCN2 adenovirus were gifted by Fibrogen, Inc, CA. After washing three times with PBST, the membranes were incubated with suitable secondary antibodies for 1 hour at room temperature. Following three additional washes with PBST, the membranes were developed with the Vistra ECF Western blotting system (GE Health Care, Piscataway, NJ) according to the manufacturer's instructions. The membranes were then scanned using a STORM PhosphorImager (Molecular Dynamics, Sunnyvale, CA), and the band intensities were measured using ImageQuant software (GE Health Care, Piscataway, NJ) and normalized to β‐actin (as a control).

### 2.6 Immunohistology

Immunohistology was performed as described previously.^13^ Briefly, HDFs were fixed with 2% paraformaldehyde, permeabilized with 0.5% Triton X‐100 in PBS, and blocked with 5% control serum in PBS. The cells were then incubated with primary antibodies for 1 hour at room temperature, followed by secondary antibodies for an additional hour. After staining, the slides were examined using a digital imaging microscope (Zeiss, Germany). Isotype‐control immunoglobulin (mouse IgG2a) was used to confirm the specificity of staining, and no detectable staining was observed with isotype‐controls.

### 2.7 [^3^H]thymidine incorporation assay

Primary HDFs were synchronized and serum stimulated, as described above. Subsequently, cells were incubated with 0.5 μCi/mL [^3^H]thymidine for 3 h. After washing, DNA was TCA precipitated, and [^3^H]DNA was quantified by scintillation counting.^24^

### 2.8 Flow cytometry

Primary HDFs were either transfected with the indicated siRNAs or not transfected as described above. The cells were then washed in PBS, and harvested with 0.05% trypsin (see above), and pelleted by centrifugation at 2000 RPM. The cells were washed again in PBS and pelleted. The pelleted cells were resuspended in 0.2 mL of PBS, then while vertexing, 1.8 mL of 70% ethanol was added dropwise, and the suspension was vortexed for an additional 2 min. The fixed cells were pelleted, and then resuspended in 1 mL of propidium iodine/RNA staining solution (0.1% Triton X‐100 in PBS, 200 μg/mL DNase‐free RNase A). ModFit software (version 4.1) was used to analyze the histograms and determine the proportion of cells in G_0_/G_1_, S, and G_2_/M phases.

### 2.9 CDK activity assays

CDK4‐cyclin D1 and CDK2‐cyclin E assays were performed as previously described.^25^, ^26^ Briefly, after transfection and synchronization of the HDFs (described above), cells were stimulated with 15% FCS for 8 h or 17 h to determine CDK4 and CDK2 activities, respectively. Lysates (100 μg) were immunoprecipitated with a polyclonal antibody to CDK4 or CDK2 (3 μg, Santa Cruz Biotechnology), as indicated. Immunoprecipitates were analyzed for the ability to phosphorylate recombinant retinoblastoma protein (pRb) (for CDK4) or histone H1 (for CDK2) in vitro, and [^32^P]phosphate incorporation was quantified using a PhosphorImagerTM (Molecular Dynamics,CA). Data are presented as %fold activation over basal level analysis.

### 2.10 Crystal violet assay

The relative density of cells was determined by crystal violet assay. Briefly, the culture medium was removed from tissue culture plate, and washed gently with PBS three times, followed by adding crystal violet solution. The plate was incubated for 10 min at room temperature and washed with tap water three times by immersion in a large beaker. The plate was drained by upside down on paper towels, then added 1% SDS to solubilize the stain. The pate then agitated on orbital shaker until color is uniform with no areas of dense coloration in bottom of wells. Upon solubilization, the amount of dye taken up by the cells was quantitated in a spectrophotometer by reading absorbance of each well at 570 nm.

### 2.11 Statistical analysis

The data are presented as mean ± SEM. Statistical analysis was performed using GraphPad Prism (v.8) with unpaired two‐sided Student's *t‐*tests, one‐way analysis of variance with Tukey's method for multiple comparisons, or Kruskal–Wallis test with Dunn's multiple comparisons test. Statistical significance was defined as *p* < 0.05. All experiments were repeated a minimum of three times unless otherwise stated.

## 3 RESULTS

### 3.1 CCN2 is essential for primary human dermal fibroblast proliferation

**FIGURE 1:**
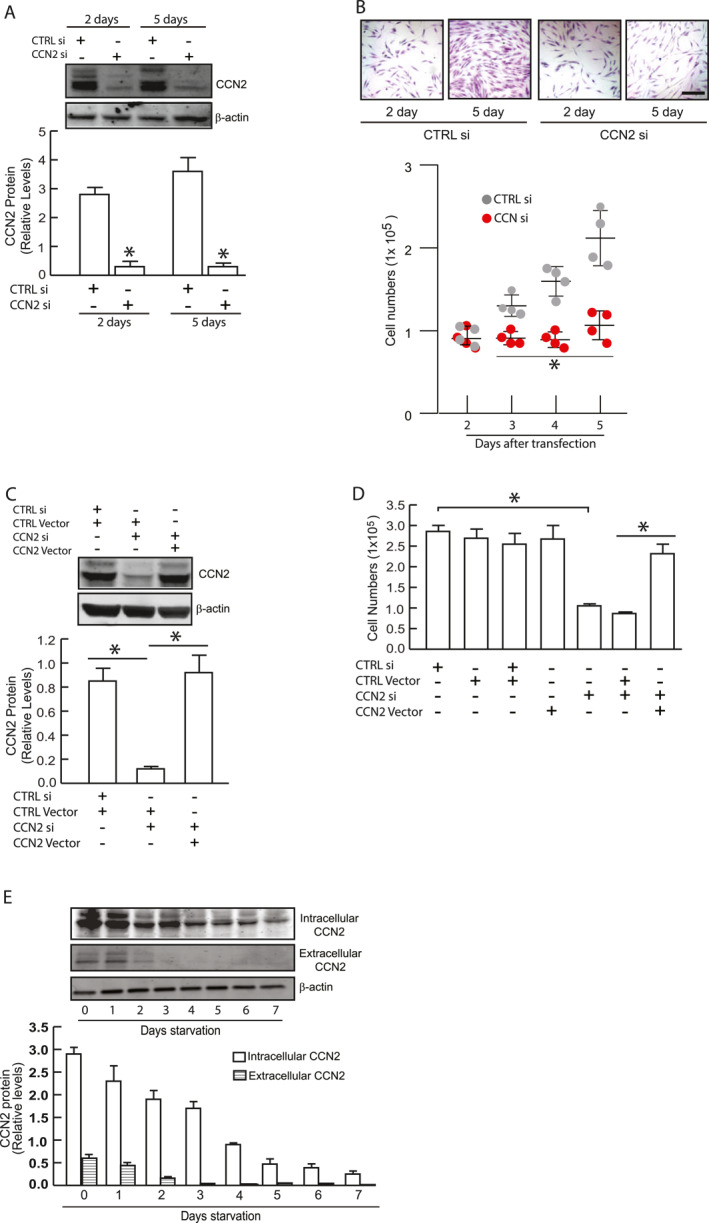
CCN2 is required for proliferation of primary HDFs. Primary HDFs were transfected with either control siRNA or CCN2 siRNA for 2 days. (A) CCN2 knockdown efficiency. CCN2 protein levels were determined by Western analysis after two or 5 days of transfection. Mean ± SEM, *N* = 3, *p < 0.05. (B) Effect of CCN2 knockdown on cell proliferation. x10^5^ cells were plated in 35 mm dishes. Cells were harvest and counted at indicated days. Crystal violet staining is shown for two‐ and 5‐days incubation. Mean ± SEM, *N* = 4, *p < 0.05. Scale bars = 200 μm (C) Rescue of CCN2 expression using siRNA‐resistant CCN2. CCN2 protein levels were determined by Western analysis. Mean ± SEM, *N* = 3, *p < 0.05. (D) Rescue of cell proliferation using siRNA‐resistant CCN2. 1 × 10^5^ cells were plated in 35 mm dishes. Cell counts were performed after 5 days of culture. Mean ± SEM, *N* = 3, *p < 0.05. (E) Intracellular and secreted CCN2 protein expression with increasing cell confluence. 1.5 × 10^6^ primary HDF cells were plated in 100 mm dishes and harvested at indicated time points. CCN2 intracellular (cell) and extracellular (media) protein levels were determined by Western analysis. Mean ± SEM, *N* = 3. Protein levels were normalized to β‐actin (used as a loading control). Representative Western blot images are shown as insets. HDFs, human dermal fibroblasts.

During our earlier research on the effects of CCN2 knockdown on collagen production, we noted a significant decrease in cell proliferation in primary HDFs.^13^ To validate and expand upon our initial findings, we assessed the impact of CCN2 knockdown (Figure 1A) on primary HDF proliferation over 5‐day period. Proliferating fibroblasts were significantly inhibited following CCN2 knockdown (Figure 1B). Notably, the reduction in cell proliferation was reversed by co‐transfection of a plasmid containing CCN2 expression cassette with two silent mutations, rendering it resistant to siRNA knockdown (Figure 1C,D). Furthermore, we observed that both intracellular and secreted CCN2 protein levels decreased significantly as cells reached confluence and stopped proliferating (Figure 1E). Collectively, these results demonstrate that CCN2 is essential for the proliferation of primary HDFs.

### 3.2 CCN2 plays a critical role in cell cycle progression in human dermal fibroblasts

**FIGURE 2:**
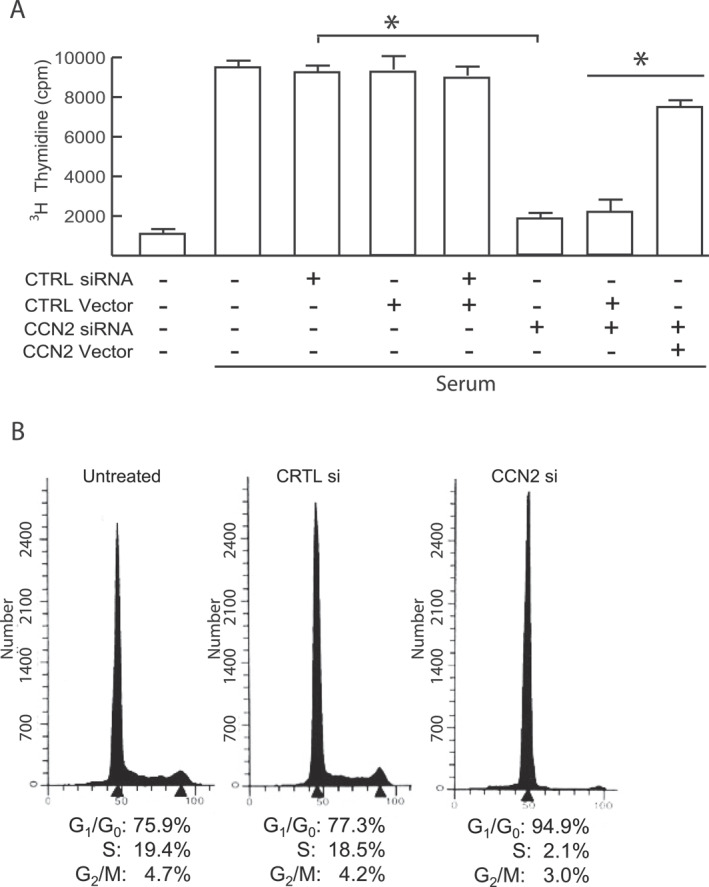
CCN2 knockdown induces G1 cell cycle arrest. (A) CCN2 is essential for DNA synthesis. Primary HDFs were transfected with the indicated siRNAs and/or a CCN2 siRNA‐resistant expression plasmid (CCN2 vector) where noted. Cells were synchronized in G0 by serum starvation for 4 days, then stimulated with 15% FCS for 20 h. Subsequently, cells were incubated with 0.5 μCi/mL [^3^H]thymidine for 3 h and [^3^H]DNA was quantified by scintillation counting. Mean ± SEM, *N* = 3, *p < 0.05. (B) CCN2 depletion leads to G1 cell cycle arrest. Cells were either transfected with the indicated siRNA or left untreated. Asynchronous cells were analyzed by flow cytometry and phase percentages are indicated on each chart. The histogram shown is representative of three independent experiments. HDFs, human dermal fibroblasts; FCS, fetal calf serumfetal calf serum.

We examined the HDF cell cycle profile in which CCN2 knockdown inhibited proliferation. Primary HDFs were arrested in G0 by serum starvation and then stimulated with 15% FCS to re‐enter the cell cycle synchronously. CCN2 siRNA knockdown blocked entry of HDFs into S‐phase (Figure 2A). Importantly, DNA synthesis was rescued by co‐transfection of a CCN2 siRNA resistance expression vector (Figure 2A, last lane), demonstrating that the blockade of cell cycle progression is specifically due to loss of CCN2. Flow cytometry analysis supported these data, showing that CCN2 knockdown led to an accumulation of HDFs in the G1/G0 phase (Figure 2B). These data demonstrate that CCN2 knockdown in HDFs caused a significant accumulation of cells in G1/G0 phase and blocked entry into the S‐phase.

### 3.3 CCN2 knockdown affects mRNA expression of cell cycle regulators

**FIGURE 3:**
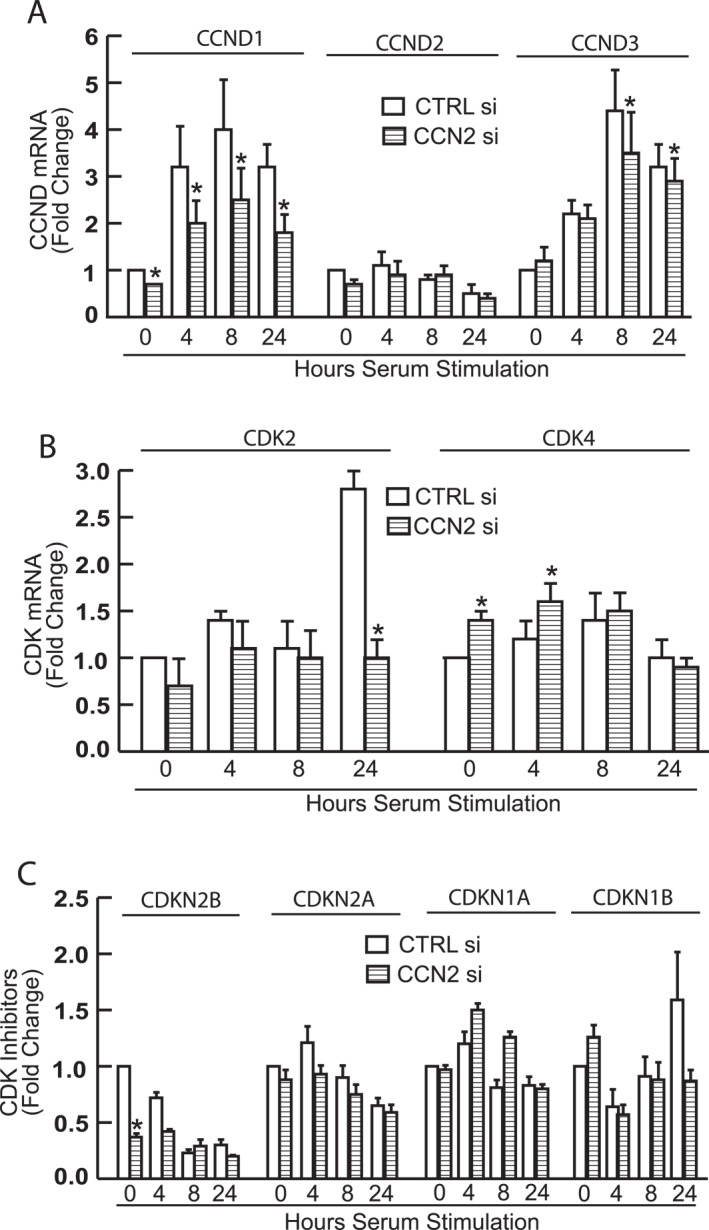
Impact of CCN2 knockdown on mRNA expression of cell cycle regulators. Primary HDF cells were transfected with either control siRNA or CCN2 siRNA for 2 days. Following transfection, cells were synchronized by serum starvation for 4 days and stimulated with 15% FCS for indicated times. mRNA expression levels were quantified using real‐time RT‐PCR for: (A) G1 cyclins, CCND (CCND1/cyclin D1, CCND2/cyclin D2, CCND3/cyclin D3); (B) Cyclin‐dependent kinases (CDKs); (C) Cyclin‐dependent kinase inhibitors (CKIs, CDKN2B/p15, CDKN2A/p16, CDKN1A/p21 CDKN1B/p27). mRNA levels were normalized to the housekeeping gene 36B4 (internal control for quantification). Data are presented as mean ± SEM, *n* = 3, *p < 0.05. HDF, human dermal fibroblast; FCS, fetal calf serumfetal calf serum.

Because HDFs depleted of CCN2 arrest in G1/G0 phase of the cycle, we examined expression of genes crucial for G1 phase progression, notably G1 cyclins, CDKs, and CDK inhibitors (CKIs). HDFs were transfected with control or CCN2 siRNA, arrested in G0 by serum deprivation, and stimulated with FCS to re‐enter the cell cycle synchronously. Unexpectedly, we found that the expression of G1 cyclins (Figure 3A), CDKs (Figure 3B), and CKIs (Figure 3C) were only modestly affected by CCN2 knockdown. Specifically, basal expression of CCND1/cyclin D1 was reduced by approximately 30% and maximum induction (8 hours after serum stimulation) reduced by 40% (Figure 3A, left). Knockdown of CCN2 led to a moderate decrease in CCND3/Cyclin D3 levels at 8‐ and 24‐h following serum stimulation (Figure 3A, right). However, CCND2/cyclin D2 was not significantly altered by knockdown of CCN2 (Figure 3A, middle), whereas both cyclin D1 and cyclin D3 exhibited the expected induction following serum stimulation. CCN2 knockdown did not significantly impact CKI levels, whereas serum stimulation led to a decrease in CDKN2B/p15 expression (Figure 3C). Taken together, these data indicate that expression of the D‐type cyclins, CDKs, and CKIs are not strongly altered by loss of CCN2, despite the G1 phase cell cycle arrest shown in Figure 2.

### 3.4 CCN2 knockdown abrogates cyclin E accumulation and CDK2 activity

**FIGURE 4:**
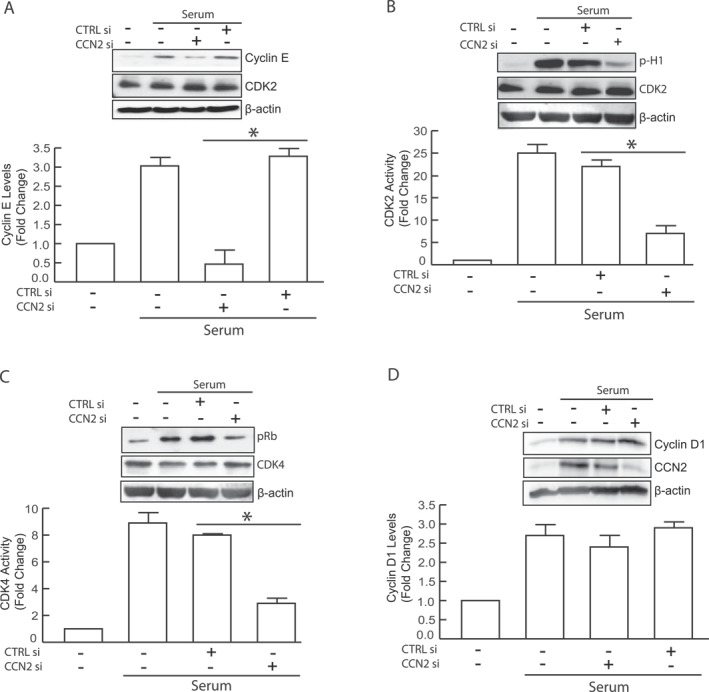
Knockdown of CCN2 decreases cyclinE‐CDK2 activity. Primary HDF cells were transfected with either control siRNA or CCN2 siRNA for 2 days. Following transfection, cells were synchronized by serum starvation for 4 days and stimulated with 15% FCS for 20 h. (A) Cyclin E and CDK2 protein levels were measured by Western analysis. (B) CDK2 activity was measured by in vitro kinase assay with histone H1 as the substrate. (C) CDK4 activity was measured by in vitro kinase assay with retinoblastoma protein as the substrate. (D) Cyclin D1 and CCN2 protein levels were measured by Western analysis. Protein levels were normalized to β‐actin (used as a loading control). Representative Western blot images are shown as insets. Data are presented as mean ± SEM, *n* = 3, *p < 0.05. HDF, human dermal fibroblast; FCS, fetal calf serumfetal calf serum.

Loss of CCN2 abrogated cells entry into the S‐phase (Figure 2). Because cyclin E expression is necessary for late G_1_ activation of CDK2,^27^, ^28^ we investigated whether CCN2 loss affects cyclin E expression. In serum starved HDFs, cyclin E expression was undetectable, but serum stimulation significantly increased its expression (Figure 4A). HDFs transfected with control siRNA showed a similar increase in cyclin E expression upon serum stimulation. However, CCN2 knockdown diminished the serum‐increased cyclin E expression. We speculated that CDK2 activity, which is dependent on cyclin E binding, would concomitantly decrease under these conditions. Using an in vitro kinase assay with histone H1 as the substrate, we found that CCN2 knockdown significantly inhibited CDK2 activity, likely due to reduced cyclin E induction (Figure 4B). The inability of HDFs to enter the S‐phase after CCN2 depletion can be explained by the insufficient accumulation and subsequent failure to activate CDK2 by cyclin E. CDK2‐cyclin E complex plays a crucial role in regulating passage through the G1 restriction point by hyper‐phosphorylation of pRb.^29^ Accordingly, we examined enzymatic activity of CDK4 and found it to be significantly impaired in cells with CCN2 knockdown (Figure 4C). Although this finding elucidates the loss of cyclin E accumulation in these cells, it was somewhat unexpected given that CCN2 knockdown did not impair induction of D‐type cyclins and CDK4 gene expression (Figure 3). Indeed, accumulation of the inherently instable cyclin D proteins predominantly regulated by post‐transcriptional and translational mechanisms. We therefore hypothesized that impaired cyclin D accumulation might underlie the loss of CDK4 activity. Contrary to this hypothesis, we found that CCN2 knockdown does not affect the accumulation of cyclin D1 protein following serum stimulation (Figure 4D). Collectively, these results demonstrate that CCN2 is essential for cyclin E expression, and that the impaired cell cycle progression observed after CCN2 depletion can be attributed to the loss of CDK2‐cyclin E activity.

### 3.5 CCN2 knockdown blocks CDK4‐cyclin D nuclear translocation

**FIGURE 5:**
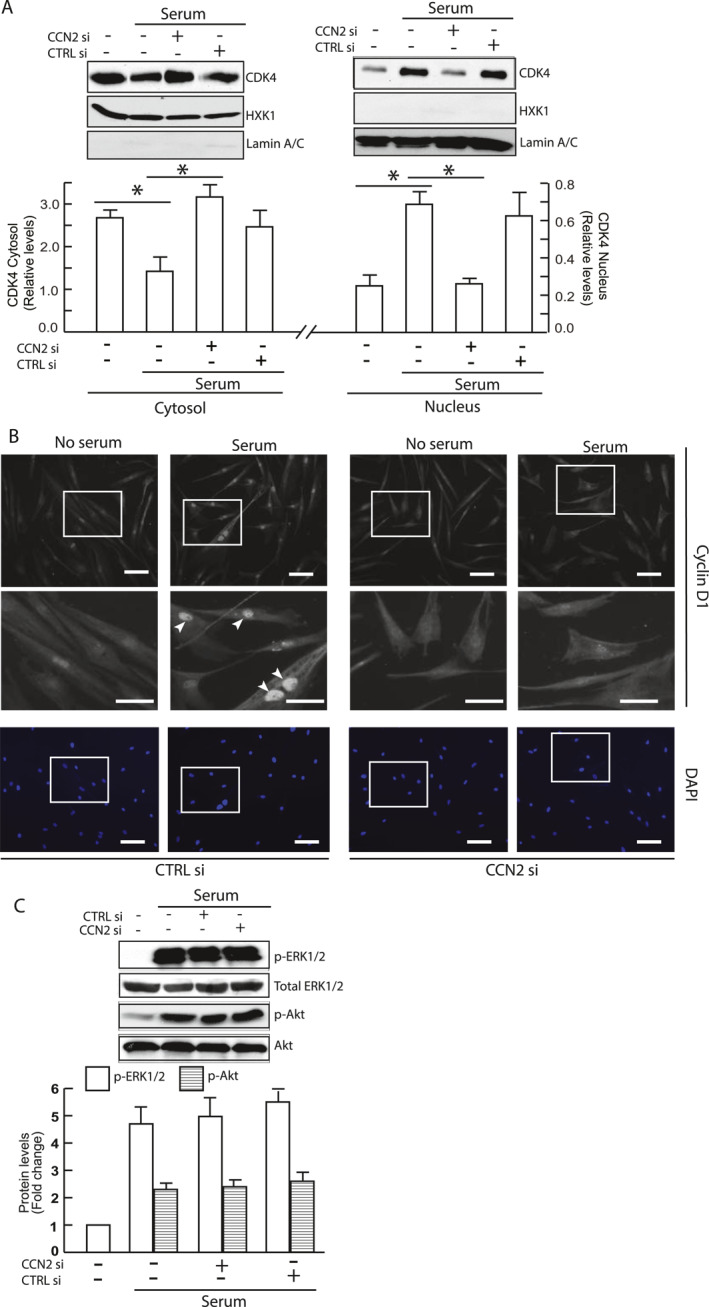
CCN2 regulates cyclin D1‐CDK4 nuclear translocation. Primary HDF cells were transfected with either control siRNA or CCN2 siRNA for 2 days. Cells were then synchronized by serum starvation for 4 days, followed by stimulation with 15% FCS for 20 h. (A) CDK4 protein levels in cytosolic (HXK1as marker) and nuclear (lamin A/C as marker) fractions were assessed by Western blot analysis. (B) Cyclin D1 nuclear translocation was visualized by immunofluorescence. A 2.5x magnification of the highlighted area is displayed in the middle panel. Nuclei are shown in blue (bottom panel). Images are representative of four independent experiments. Scale bars = 100 μm. (C) ERK and Akt phosphorylation levels were determined by Western blot analysis. Protein levels were normalized to β‐actin (loading control). Insets show representative Western blot images. Data are presented as mean ± SEM, *N* = 3, *p < 0.05. HDF, human dermal fibroblast; FCS, fetal calf serumfetal calf serum.

Impaired CDK4 activity despite normal expression of CDK4 and D‐type cyclins suggests that CCN2 knockdown may affect nuclear translocation of the cyclin D‐CDK4 complex. CDK activation occurs in the nucleus and is regulated by T‐loop phosphorylation (CDK activating kinase) and dephosphorylation (Cdc25 phosphatase). To investigate this, we examined nuclear translocation of cyclin D‐CDK4 complexes using cell fractionation and immunofluorescence. Our findings demonstrate that CCN2 knockdown inhibits nuclear translocation of CDK4 and cyclin D (Figure 5). Western blotting revealed serum stimulation decreased cytosolic CDK4 (Figure 5A, left panel) while increasing nuclear CDK4 (Figure 5A, right panel). CCN2 knockdown significantly reduced this response. Immunofluorescence corroborated these results, revealing cyclin D accumulation in the nucleus following serum stimulation (Figure 5B, middle left), which was largely impaired by CCN2 knockdown (Figure 5B, right). The impairment nuclear translocation of cyclin D‐CDK4 explains the reduced cyclin E accumulation observed after CCN2 knockdown. This is because the primary target of CDK4, pRb, is predominantly nuclear. Our previous work has shown that activation of the RAF‐MEK‐ERK cascade is necessary for the nuclear translocation of CDK2‐cyclin E complexes.^26^ Given this, we investigated the MAPK pathway to understand the mechanism underlying the impaired nuclear translocation of CDK4‐cyclin D following CCN2 knockdown. Interestingly, we observed that ERK and AKT phosphorylation were unaffected by CCN2 knockdown (Figure 5C). This observation aligns with our expectations, as disruption of either pathway would typically lead to reduced cyclin D1 accumulation, which we did not observe in our previous experiments (Figure 4D).

### 3.6 CCN2 translocation to the nucleus and co‐localizes with cyclin D1

**FIGURE 6:**
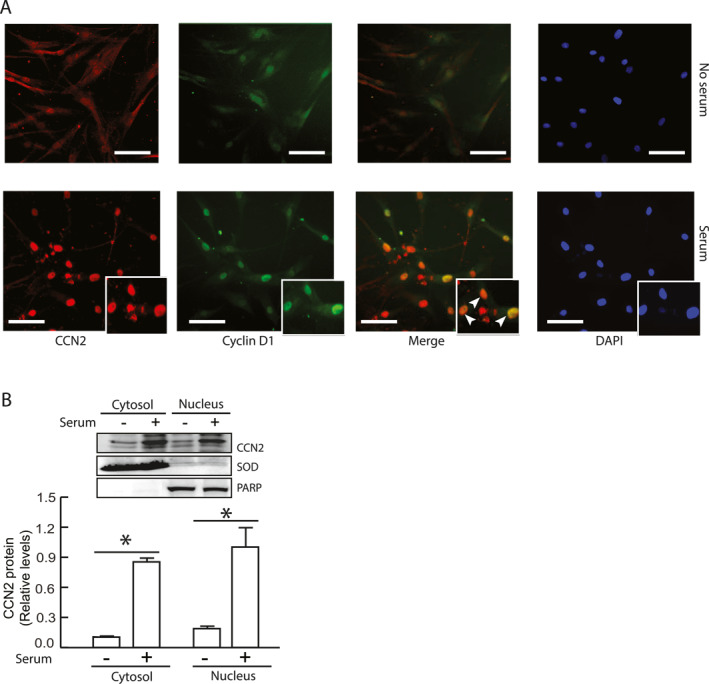
CCN2 translocates to the nucleus and co‐localizes with cyclin D1. Primary HDF cells were synchronized by serum starvation for 4 days, followed by stimulation with 15% FCS for 20 h. (A) CCN2 (left panel) and cyclin D1 (middle left panel) nuclear translocation were visualized by immunofluorescence. Boxed areas are shown at 2.5x magnification as insets. CCN2 and cyclin D1 merged panel shown (middle right panel). DAPI staining (right panel) delineates nuclei. Images are representative of three independent experiments. Scale bars = 100 μm. (B) CCN2 protein levels in cytosolic (SOD as marker) and nuclear (PARP as marker) fractions were assessed by Western blot analysis. Protein levels were normalized to β‐actin (loading control). Insets show representative Western blot images. Data are presented as mean ± SEM, *N* = 3, *p < 0.05. HDF, human dermal fibroblast; FCS, fetal calf serumfetal calf serum.

To further investigate how CCN2 might influence the nuclear accumulation of cyclin D complexes in early G1 phase, we examined whether CCN2 co‐localizes with cyclin D‐CDK4 complexes and affects their subcellular localization. Using double immunofluorescence staining, we unexpectedly observed that CCN2 translocated to the nucleus (Figure 6A, left panel) and co‐localized with cyclin D1 (Figure 6, middle right panel) upon serum stimulation. Western blotting further confirmed that subset CCN2 protein indeed translocated into nucleus upon serum stimulation (Figure 6B). Collectively, these data indicate that CCN2 interacts with cyclin D‐CDK4 complexes and facilitates their nuclear entry, a function essential for the proliferation of primary HDFs.

### 3.7 CCN2 mediates YAP/TAZ‐dependent proliferation of primary human dermal fibroblasts

**FIGURE 7:**
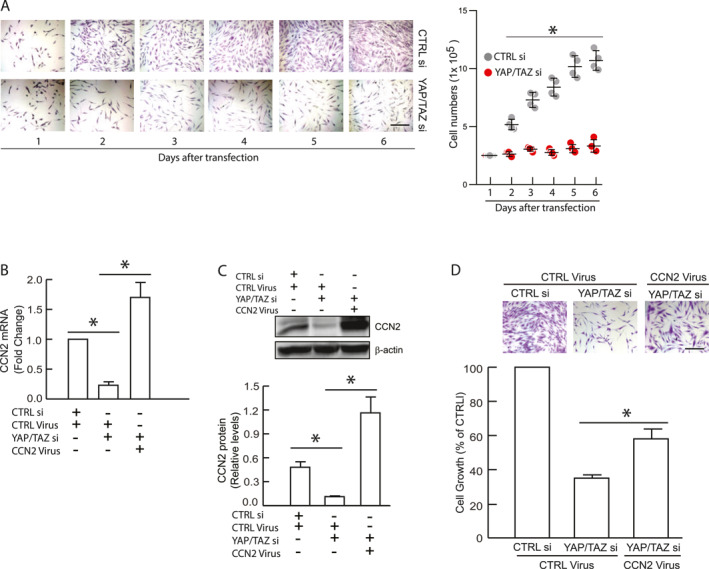
CCN2 partially mediates YAP/TAZ‐dependent proliferation. (A) YAP/TAZ knockdown inhibits HDF proliferation. Cells were transfected with either control siRNA or YAP/TAZ siRNAs for 2 days. 2 × 10^5^ cells were cultured in 10 cm plates for the indicated days and counted. Cells were visualized by crystal violet staining. Images are representative of four independent experiments. Results are expressed as mean ± SEM, *N* = 4. Scale bars = 200 μm. (B, C and D) cells were transfected with the indicated siRNA and infected with indicated adenovirus for 2 days. CCN2 mRNA (B) and protein (C) levels were determined by real‐time PCR and Western blot analysis, respectively. mRNA levels were normalized to 36B4, a ribosomal protein used as an internal control. Protein levels were normalized to β‐actin as a loading control. Insets show representative Western blots. Results are expressed as mean ± SEM, *N* = 4, *p < 0.05. (D) CCN2 mediates YAP/TAZ‐dependent cell proliferation. 2 × 10^5^ cells were cultured in 10 cm plates for 6 days, then stained with crystal violet blue dye and quantified. Images are representative of four independent experiments. Results are expressed as mean ± SEM, *N* = 4, *p < 0.05. Scale bars = 200 μm. HDF, human dermal fibroblast; TAZ, transcriptional coactivator with PDZ‐binding motif; YAP, yes‐associated protein.

We explored the relationship between CCN2 and YAP/TAZ in the context of cell proliferation, as CCN2 is a bona fide YAP/TAZ target gene, and YAP/TAZ critically regulates cell proliferation in HDFs.^14^, ^23^, ^30^ Knockdown of YAP/TAZ significantly inhibited cell proliferation, and almost completely blocking the proliferation of primary HDFs (Figure 7A). To investigate whether CCN2 mediates YAP/TAZ‐dependent cell proliferation, we restored CCN2 expression in YAP/TAZ knockdown cells using a CCN2 adenovirus (Figures 7B,C). CCN2 restoration partially, but significantly, reversed YAP/TAZ‐dependent inhibition of HDFs proliferation (Figure 7D). These data suggest that CCN2 partially mediates YAP/TAZ‐dependent cell proliferation in primary HDFs.

## 4 DISCUSSION

Although CCN2 is traditionally acknowledged as a profibrotic mediator involved in fibrotic disorders through its impact on collagenous ECM regulation, our research uncovers an additional crucial function. We demonstrate that CCN2 plays a vital role in controlling the proliferation of primary HDFs by modulating cell cycle progression.

CCN2 knockdown resulted in a significant inhibition of HDF proliferation, which was reversible upon CCN2 restoration by a siRNA‐resistant CCN2 expression vector. This effect was accompanied by G1/G0 phase arrest, indicating that CCN2 is essential for the G1/S phase transition and elucidate a novel mechanism by which CCN2 facilitates this process. Unexpectedly, the expression of major G1 phase regulators, including cyclins, CDKs, and CKIs, was only modestly affected by CCN2 depletion. This suggests that the role of CCN2 in cell cycle regulation extends beyond transcriptional control of these factors.

Cells are stimulated into the cell cycle from quiescence by mitogenic signaling, which is sensed by expression of D‐type cyclins that preferentially bind and activate CDK4 during G_1_.^18^ Activation of cyclin D‐CDK4 complexes leads to partial inactivation of pocket proteins pRB, p107, and p130, which induces expression of E‐type cyclins that bind and activate CDK2. Activated cyclin E‐CDK2 complexes further phosphorylate the pocket proteins, which propel cells through the G_1_/S restriction point.^27^ A key finding of our study is the abrogation of cyclin E accumulation and CDK2 activity following CCN2 knockdown. Cyclin E is essential for late G1 activation of CDK2 and subsequent S‐phase entry. The impaired cyclin E expression explains the G1/S transition block observed in CCN2‐depleted cells. Cyclin E‐CDK2 activity is in part‐regulated upstream by cyclin D1‐CDK4 activity. However, the pathways that lead to cyclin D accumulation, as well as cyclin D1 levels, are normal in CCN2 siRNA treated cells (Figure 3A), suggesting that the observed cell cycle arrest is due to a decrease in cyclin D1‐CDK4 activity. In mammalian cells, cyclinD‐CDK4 is the first kinase activated by the mitogenic signals that release cells from the quiescent or G_0_ arrested state.^31^ Importantly, this effect was traced back to reduced CDK4 activity, despite normal expression and accumulation of cyclin D1 protein. Further investigation revealed that CCN2 knockdown impairs the nuclear translocation of cyclin D1‐CDK4 complexes. This finding is particularly significant as it provides a mechanistic explanation for the reduced CDK4 activity and subsequent cyclin E accumulation failure. Interestingly, CCN2 depletion did not affect ERK and AKT phosphorylation, ruling out these pathways as the cause of impaired nuclear translocation.

The most striking discovery is the nuclear translocation of CCN2 itself and its co‐localization with cyclin D1 upon serum stimulation. This observation suggests a direct role for CCN2 in facilitating the nuclear entry of cyclin D1‐CDK4 complexes, a function that appears to be essential for HDF proliferation. Nuclear translocation of CCN2 to regulate cell cycle is surprising because the mechanism involved in the nuclear import of cyclin D complexes is thought to be well understood.^18^ Although neither D‐cyclins nor CDK4/6 contain a nuclear localization signal (NLS), nuclear translocation is normally achieved through the NLS found on p21, a CKI that forms a ternary complex with cyclin D and CDK4/6. To our knowledge, this is the first report of CCN2 appearing in the nucleus of HDF cells. Our results hint that CCN2 may co‐localize and interact with cyclin D‐CDK in a way that affects its subcellular localization. Nonetheless, additional studies are warranted to uncover the precise molecular mechanism(s) by which CCN2 regulates cyclin D nuclear translocation.

Our results also shed light on the relationship between CCN2 and the YAP/TAZ pathway in regulating HDF proliferation. When CCN2 was reintroduced after YAP/TAZ knockdown, it partially reversed the decrease in proliferation, suggesting that CCN2 plays a role in facilitating YAP/TAZ‐driven cell division in HDFs. This finding underscores the importance of CCN2 in integrating various signaling pathways that control fibroblast proliferation. YAP and TAZ are major downstream effector of the hippo signaling pathway, which was originally recognized as a vital regulator of organ size in animals.^30^, ^32^ Little is known regarding the functional significance of YAP/TAZ in skin dermal fibroblasts. Moreover, although CCN2 is a well‐known bona fide YAP/TAZ target gene,^14^, ^23^, ^33^ its role in mediating YAP/TAZ functions is poorly understand in HDFs. We demonstrate that in human skin dermal fibroblast, knockdown of YAP/TAZ significantly reduced the expression of CCN2 and completely repressed proliferation. We previously reported that in human keratinocytes, knockdown of YAP/TAZ significantly reduced the expression of both CCN2 and CCN1, a first member of CCN family protein, and inhibited proliferation and survival.^23^ This keratinocyte inhibition of proliferation is near completely rescued by restoration of CCN1 expression, but not by CCN2 expression. These data demonstrate that YAP/TAZ‐dependent fibroblast and keratinocyte proliferations are mediated by CCN2 and CCN1, respectively. These data suggest that CCN1 and CCN2 proteins function as YAP/TAZ downstream mediator and might play an important role in the maintenance of skin epidermal and dermal homeostasis. Additionally, YAP/TAZ and their downstream target CCN1 and CCN2 are markedly elevated in human basal cell carcinoma (BCC).^23^ These data suggest YAP/TAZ contributes to BCC formation through up‐regulation of CCN1 and CCN2, which promote keratinocyte growth and survival and stromal cell activation and proliferation, respectively. Targeting YAP and/or CCN1 and CCN2 may provide clinical benefit in BCC and other cancers in which YAP is elevated.

Mechanical forces play a crucial role in cellular behavior, and YAP/TAZ have emerged as key mediators in this process, known as mechanotransduction.^34^, ^35^ These proteins translate physical cues into biochemical signals and gene expression changes. The activity of YAP/TAZ is influenced by ECM properties, including stiffness and composition, allowing cells to detect and respond to their mechanical environment. CCN2 has been identified as a critical downstream target of YAP/TAZ in the mechanotransduction pathway.^34^, ^35^ It serves to amplify and transmit mechanical signals, modify the ECM, affect cell‐ECM interactions, and regulate various cellular processes in response to mechanical stimuli. One of well‐known functions of CCN2 is to promote the production of collagenous ECM, which can increase tissue stiffness. This stiffening may, in turn, further activate YAP/TAZ, potentially establishing a positive feedback loop. This mechanism is particularly significant in the context of fibrotic diseases. Our research suggests the possibility that CCN2, as a crucial downstream effector of YAP/TAZ in mechanotransduction, may also function as a cell cycle regulator. Specifically, we propose that it could stimulate the proliferation of dermal fibroblasts in response to YAP/TAZ‐mediated mechanotransduction. However, it is important to note that further investigation is necessary to elucidate the precise mechanism by which CCN2 promotes cell cycle progression in response to YAP/TAZ‐dependent mechanotransduction.

Our findings have important implications for our understanding of fibroblast biology and potential therapeutic approaches. The central role of CCN2 in fibroblast proliferation suggests that it could be a valuable target for treating fibrosis‐related disorders or promoting wound healing. Moreover, the newly discovered nuclear function of CCN2 opens new avenues for research into the multifaceted roles of this protein in cellular processes. Future studies should aim to elucidate the precise mechanism by which CCN2 facilitates the nuclear translocation of cyclin D‐CDK4 complexes and investigate whether this function is conserved in other cell types. Additionally, exploring the interplay between CCN2 and other signaling pathways involved in cell cycle regulation could provide a more comprehensive understanding of fibroblast proliferation control.

In summary, our research study reveals a new function of CCN2 as a critical regulator of the cell cycle in HDFs. CCN2 knockdown led to an accumulation of cells in the G1/G0 phase and prevented entry into S‐phase by blocking cyclin E and CDK4/cyclin D nuclear translocation and CDK2 activity. CCN2 also translocated to the nucleus and co‐localized with cyclin D1 upon cell cycle stimulation. Furthermore, CCN2 partially mediates YAP/TAZ‐dependent proliferation of primary HDFs as a bona fide target gene of YAP/TAZ. This finding represents a novel mechanism by which CCN2 regulates cell cycle progression, extending beyond its well‐established extracellular functions.

## AUTHOR CONTRIBUTIONS

TQ, GJF, and JJB participated in conceptualization and experimental design. YS, TP, YJ, ZQ, TQ, NHL, and JJB performed the experiments and organized the data. TQ, GJF, and JJB provided the financial support. TQ prepared figures and drafted the original manuscript. GJF, NHL, and JJB reviewed and edited the manuscript. All authors read and approved the final version of the article.

## CONFLICT OF INTEREST STATEMENT

The authors declare no conflicts of interest.

## ETHICS STATEMENT

The research protocol was approved by an Institutional Review Board.

## Data Availability

All data underlying this article are present in the paper. Requests for any material in this study should be directed to the corresponding author.

## References

[1] Brigstock, D. R. , R. Goldschmeding , K. I. Katsube , S. C. Lam , L. F. Lau , K. Lyons , C. Naus , et al. 2003. “Proposal for a Unified CCN Nomenclature.” Molecular Pathology 56: 127–128. 12665631 10.1136/mp.56.2.127 PMC1187305

[2] Perbal, B. 2003. “The CCN3 (NOV) Cell Growth Regulator: A New Tool For Molecular Medicine.” Expert Review of Molecular Diagnostics 3(5): 597–604. 14510180 10.1586/14737159.3.5.597

[3] Perbal, B. 2004. “CCN Proteins: Multifunctional Signalling Regulators.” Lancet 363(9402): 62–64. 10.1016/s0140-6736(03)15172-0. 14723997

[4] Brigstock, D. R. 1999. “The Connective Tissue Growth factor/Cysteine‐Rich 61/nephroblastoma Overexpressed (CCN) Family.” Endocrine Reviews 20(2): 189–206. 10.1210/edrv.20.2.0360. 10204117

[5] Chen, C.‐C. , N. Chen , and L. F. Lau . 2001. “The Angiogenic Factors Cyr61 and Connective Tissue Growth Factor Induce Adhesive Signaling in Primary Human Skin Fibroblasts.” Journal of Biological Chemistry 276(13): 10443–10452. 10.1074/jbc.m008087200. 11120741

[6] Grotendorst, G. R. 1997. “Connective Tissue Growth Factor: a Mediator of TGF‐β Action on Fibroblasts.” Cytokine & Growth Factor Reviews 8(3): 171–179. 10.1016/s1359-6101(97)00010-5. 9462483

[7] Henrot, P. , M.‐E. Truchetet , G. Fisher , A. Taïeb , and M. Cario . 2019. “CCN Proteins as Potential Actionable Targets in Scleroderma.” Experimental Dermatology 28(1): 11–18. 10.1111/exd.13806. 30329180

[8] Holmes, A. , D. J. Abraham , S. Sa , X. Shiwen , C. M. Black , and A. Leask . 2001. “CTGF and SMADs, Maintenance of Scleroderma Phenotype Is Independent of SMAD Signaling.” Journal of Biological Chemistry 276(14): 10594–10601. 10.1074/jbc.m010149200. 11152469

[9] Liu, S. , X. Shi‐wen , D. J. Abraham , and A. Leask . 2011. “CCN2 Is Required for Bleomycin‐Induced Skin Fibrosis in Mice.” Arthritis & Rheumatism 63(1): 239–246. 10.1002/art.30074. 20936632

[10] Sonnylal, S. , X. Shi‐Wen , P. Leoni , K. Naff , C. S. Van Pelt , H. Nakamura , A. Leask , D. Abraham , G. Bou‐Gharios , and B. de Crombrugghe . 2010. “Selective Expression of Connective Tissue Growth Factor in Fibroblasts In Vivo Promotes Systemic Tissue Fibrosis.” Arthritis & Rheumatism 62(5): 1523–1532. 10.1002/art.27382. 20213804 PMC3866029

[11] Duncan, M. R. , K. S. Frazier , S. Abramson , S. Williams , H. Klapper , X. Huang , and G. R. Grotendorst . 1999. “Connective Tissue Growth Factor Mediates Transforming Growth Factor Beta‐Induced Collagen Synthesis: Down‐Regulation by cAMP.” The FASEB Journal 13: 1774–1786. 10.1096/fasebj.13.13.1774. 10506580

[12] Ivkovic, S. , B. S. Yoon , S. N. Popoff , F. F. Safadi , D. E. Libuda , R. C. Stephenson , A. Daluiski , and K. M. Lyons . 2003. “Connective Tissue Growth Factor Coordinates Chondrogenesis and Angiogenesis during Skeletal Development.” Development 130(12): 2779–2791. 10.1242/dev.00505. 12736220 PMC3360973

[13] Quan, T. , T. He , S. Kang , J. J. Voorhees , and G. J. Fisher . 2002. “Connective Tissue Growth Factor: Expression in Human Skin In Vivo and Inhibition by Ultraviolet Irradiation.” Journal of Investigative Dermatology 118(3): 402–408. 10.1046/j.0022-202x.2001.01678.x. 11874477

[14] Qin, Z. , T. He , C. Guo , and T. Quan . 2022. “Age‐Related Downregulation of CCN2 Is Regulated by Cell Size in a YAP/TAZ‐Dependent Manner in Human Dermal Fibroblasts: Impact on Dermal Aging.” JID Innovations 2(3): 100111. 10.1016/j.xjidi.2022.100111. 35480397 PMC9035808

[15] Quan, T. , Y. Shao , T. He , J. J. Voorhees , and G. J. Fisher . 2010. “Reduced Expression of Connective Tissue Growth Factor (CTGF/CCN2) Mediates Collagen Loss in Chronologically Aged Human Skin.” Journal of Investigative Dermatology 130(2): 415–424. 10.1038/jid.2009.224. 19641518 PMC2877594

[16] Igarashi, A. , H. Okochi , D. M. Bradham , and G. R. Grotendorst . 1993. “Regulation of Connective Tissue Growth Factor Gene Expression in Human Skin Fibroblasts and during Wound Repair.” Molecular Biology of the Cell 4(6): 637–645. 10.1091/mbc.4.6.637. 8374172 PMC300970

[17] Quan, T. , Y. Shao , T. He , J. J. Voorhees , and G. J. Fisher . 2010. “Reduced Expression of Connective Tissue Growth Factor (CTGF/CCN2) Mediates Collagen Loss in Chronologically Aged Human Skin.” Journal of Investigative Dermatology 130(2): 415–424. 10.1038/jid.2009.224. 19641518 PMC2877594

[18] Jamasbi, E. , M. Hamelian , M. A. Hossain , and K. Varmira . 2022. “The Cell Cycle, Cancer Development and Therapy.” Molecular Biology Reports 49(11): 10875–10883. 10.1007/s11033-022-07788-1. 35931874

[19] Abreu Velez, A. M. , and M. S. Howard . 2015. “Tumor‐suppressor Genes, Cell Cycle Regulatory Checkpoints, and the Skin.” North American Journal of Medical Sciences 7(5): 176–188. 10.4103/1947-2714.157476. 26110128 PMC4462812

[20] Bertoli, C. , J. M. Skotheim , and R. A. M. de Bruin . 2013. “Control of Cell Cycle Transcription during G1 and S Phases.” Nature Reviews Molecular Cell Biology 14(8): 518–528. 10.1038/nrm3629. 23877564 PMC4569015

[21] Fisher, G. J. , P. A. Henderson , J. J. Voorhees , and J. J. Baldassare . 1991. “Epidermal Growth Factor‐Induced Hydrolysis of Phosphatidylcholine by Phospholipase D and Phospholipase C in Human Dermal Fibroblasts.” Journal of Cellular Physiology 146(2): 309–317. 10.1002/jcp.1041460216. 1999479

[22] Gorges, L. L. , N. H. Lents , and J. J. Baldassare . 2008. “The Extreme COOH Terminus of the Retinoblastoma Tumor Suppressor Protein pRb Is Required for Phosphorylation on Thr‐373 and Activation of E2F.” American Journal of Physiology: Cell Physiology 295(5): C1151–C1160. 10.1152/ajpcell.00300.2008. 18768921

[23] Quan, T. , Y. Xu , Z. Qin , P. Robichaud , S. Betcher , K. Calderone , T. He , T. M. Johnson , J. J. Voorhees , and G. J. Fisher . 2014. “Elevated YAP and its Downstream Targets CCN1 and CCN2 in Basal Cell Carcinoma: Impact on Keratinocyte Proliferation and Stromal Cell Activation.” American Journal Of Pathology 184(4): 937–943. 10.1016/j.ajpath.2013.12.017. 24485923 PMC3969992

[24] Phillips‐Mason, P. J. , D. M. Raben , and J. J. Baldassare . 2000. “Phosphatidylinositol 3‐kinase Activity Regulates Alpha ‐Thrombin‐Stimulated G1 Progression by its Effect on Cyclin D1 Expression and Cyclin‐dependent Kinase 4 Activity.” Journal of Biological Chemistry 275(24): 18046–18053. 10.1074/jbc.m909194199. 10749883

[25] Keenan, S. M. , C. Bellone , and J. J. Baldassare . 2001. “Cyclin‐dependent Kinase 2 Nucleocytoplasmic Translocation Is Regulated by Extracellular Regulated Kinase.” Journal of Biological Chemistry 276(25): 22404–22409. 10.1074/jbc.m100409200. 11304535

[26] Lents, N. H. , S. M. Keenan , C. Bellone , and J. J. Baldassare . 2002. “Stimulation of the Raf/MEK/ERK Cascade Is Necessary and Sufficient for Activation and Thr‐160 Phosphorylation of a Nuclear‐Targeted CDK2.” Journal of Biological Chemistry 277(49): 47469–47475. 10.1074/jbc.m207425200. 12359725

[27] Hochegger, H. , S. Takeda , and T. Hunt . 2008. “Cyclin‐dependent Kinases and Cell‐Cycle Transitions: Does One Fit All?” Nature Reviews Molecular Cell Biology 9(11): 910–916. 10.1038/nrm2510. 18813291

[28] Pagano, M. , R. Pepperkok , F. Verde , W. Ansorge , and G. Draetta . 1992. “Cyclin A Is Required at Two Points in the Human Cell Cycle.” The EMBO Journal 11(3): 961–971. 10.1002/j.1460-2075.1992.tb05135.x. 1312467 PMC556537

[29] Ezhevsky, S. A. , A. Ho , M. Becker‐Hapak , P. K. Davis , and S. F. Dowdy . 2001. “Differential Regulation of Retinoblastoma Tumor Suppressor Protein by G(1) Cyclin‐dependent Kinase Complexes In Vivo.” Molecular and Cellular Biology 21(14): 4773–4784. 10.1128/mcb.21.14.4773-4784.2001. 11416152 PMC87164

[30] Zhao, B. , K. Tumaneng , and K.‐L. Guan . 2011. “The Hippo Pathway in Organ Size Control, Tissue Regeneration and Stem Cell Self‐Renewal.” Nature Cell Biology 13(8): 877–883. 10.1038/ncb2303. 21808241 PMC3987945

[31] Blain, S. 2008. “Switching Cyclin D‐Cdk4 Kinase Activity on and off.” Cell Cycle 7: 892–898. 10.4161/cc.7.7.5637. 18414028

[32] Justice, R. W. , O. Zilian , D. F. Woods , M. Noll , and P. J. Bryant . 1995. “The Drosophila Tumor Suppressor Gene Warts Encodes a Homolog of Human Myotonic Dystrophy Kinase and Is Required for the Control of Cell Shape and Proliferation.” Genes & Development 9(5): 534–546. 10.1101/gad.9.5.534. 7698644

[33] Wang, N. , X. Xu , F. Guan , Y. Zheng , Y. Shou , T. Xu , G. Shen , et al. 2024. “Alpha‐Catenin Promotes Dermal Fibroblasts Proliferation and Migration during Wound Healing via FAK/YAP Activation.” The FASEB Journal 38(2): e23410. 10.1096/fj.202302251r. 38193545

[34] Dupont, S. , L. Morsut , M. Aragona , E. Enzo , S. Giulitti , M. Cordenonsi , F. Zanconato , et al. 2011. “Role of YAP/TAZ in Mechanotransduction.” Nature 474(7350): 179–183. 10.1038/nature10137. 21654799

[35] Halder, G. , S. Dupont , and S. Piccolo . 2012. Transduction of Mechanical and Cytoskeletal Cues by YAP and TAZ. Nature Reviews Molecular Cell Biology 13(9): 591–600. 10.1038/nrm3416. 22895435

